# Breath-centered virtual mind-body medicine reduces COVID-related stress in women healthcare workers of the Regional Integrated Support for Education in Northern Ireland: a single group study

**DOI:** 10.3389/fpsyt.2023.1199819

**Published:** 2023-06-12

**Authors:** Patricia L. Gerbarg, Felicity Dickson, Vincent A. Conte, Richard P. Brown

**Affiliations:** ^1^Department of Psychiatry, New York Medical College, Valhalla, NY, United States; ^2^Regional Integrated Support for Education, Belfast, United Kingdom; ^3^Management Department, Hofstra University, Hempstead, NY, United States; ^4^Columbia University Vagelos College of Physicians and Surgeons, New York, NY, United States

**Keywords:** COVID-19 pandemic, occupational stress, healthcare workers, mind-body medicine, breathing exercises, autonomic nervous system, children with disabilities, psychological stress

## Abstract

**Background:**

During the COVID-19 pandemic, healthcare workers endured prolonged stress affecting their psychological well-being. Objectives: (1) Evaluate the effects of the Breath-Body-Mind Introductory Course (BBMIC) on COVID-related stress among employees of the Regional Integrated Support for Education, Northern Ireland, (2) Reduce the risk of adverse effects from COVID-related stress, and (3) Evaluate the effects of BBMIC on indicators of psychophysiological states and the consistency with hypothesized mechanisms of action.

**Methods:**

In this single group study, a convenience sample of 39 female healthcare workers completed informed consent and baseline measures: Perceived Stress Scale (PSS), Stress Overload Scale-Short (SOS-S), and Exercise-Induced Feelings Inventory (EFI). Following the online BBMIC 4 h/day for 3 days and the 6 week solo (20 min/day) and group practice (45 min weekly), repeat testing plus the Indicators of Psychophysiological State (IPSS) and Program Evaluation were obtained.

**Results:**

Baseline (T1) mean PSS score was significantly elevated compared to a normative sample: PSS = 18.2 vs. 13.7 (*p* < 0.001) and improved significantly 11 weeks post-BBMIC (T4). SOS-S mean score declined from 10.7(T1) to 9.7 at 6 week post-test (T3). The SOS-S proportion of High Risk scores found in 22/29 participants (T1), dropped to 7/29 (T3). EFI mean subscale scores improved significantly from T1 to T2 and T3 for Revitalization (*p* < 0.001); Exhaustion (*p* < 0.002); and Tranquility (*p* < 0.001); but not Engagement (*p* < 0.289).

**Conclusion:**

Among RISE NI healthcare workers affected by COVID-related stress, participation in the BBMIC significantly reduced scores for Perceived Stress, Stress Overload, and Exhaustion. EFI Revitalization and Tranquility scores significantly improved. More than 60% of participants reported moderate to very strong improvements in 22 indicators of psychophysiological state, e.g., tension, mood, sleep, mental focus, anger, connectedness, awareness, hopefulness, and empathy. These results are consistent with the hypothesized mechanisms of action whereby voluntarily regulated breathing exercises change interoceptive messaging to brain regulatory networks that shift psychophysiological states of distress and defense to states of calmness and connection. These positive findings warrant validation in larger, controlled studies to extend the understanding of how breath-centered Mind-body Medicine practices could mitigate adverse effects of stress.

## Introduction

During mass disasters, such as the COVID-19 pandemic, the number of people needing mental health support far exceeds the capacity of existing conventional healthcare resources using the model of one provider for one patient at a time. Pandemics place heavy burdens on already strained healthcare services (1, 2). Rapidly effective, inexpensive, low-risk, non-stigmatizing group interventions are needed to ameliorate adverse effects of COVID-related stress on the wellbeing and work efficacy of health workers (3–5). When healthcare workers experience prolonged severe stress, reactive changes in their stress response systems can impair their abilities to work effectively and make them vulnerable to cumulative emotional and physical impairments, deterioration of relationships, and professional burnout. In effect, they may become locked into a defensive psychophysiological state of feeling unsafe, anxious, overwhelmed, and exhausted (1, 6).

This study evaluated the effects of Breath-Body-Mind Introductory Course (BBMIC), a breath-centered Mind-body Medicine program, on indicators of stress and psychophysiological state among employees of the Regional Integrated Support for Education in Northern Ireland (RISE NI) during the COVID-19 pandemic, from December 16, 2020 to April 14, 2021. RISE NI is a Health and Social Care Trust (HSCT), funded by the Department of Education through the Department of Health. Approximately 100 RISE staff provide direct support for about 100,000 at-risk and special needs children, mainly ages 3 to 8 years, who are mainstreamed into public primary schools, nurseries, and playgroups. Each HSCT has a RISE Team of speech and language therapists, occupational therapists, physiotherapists, clinical psychologists, social workers, behavior therapists, and therapy assistants. RISE uses a Transdisciplinary model working across professional boundaries. The key aims are: (1) to reduce underachievement by optimizing children’s access to learning within the educational environment and (2) to foster health, well-being and social inclusion and improve the life chances of children.

The adverse effects of the COVID pandemic on learning, physical health and mental health are most severe in children already at risk for educational disparities: students of color, English as a second language learners, children from low-income households, and those with disabilities or autism spectrum disorder (7–9). During the pandemic, most services withdrew from face-to-face work with children and families. Nevertheless, many RISE staff were required to continue face-to-face support because virtual support was ineffective or inaccessible. Staff underwent changes in responsibilities due to the Health Care response to COVID. Many were deployed to acute hospital COVID wards covering jobs outside their established remit. The remaining staff had added responsibilities, including support for community services hardest hit by the loss of other healthcare services.

A review of 18 clinical studies of mind-body modalities in health workers affected by the COVID-19 pandemic found that combining mental focus, controlled breathing, and body movements to relax the body and mind had significant positive effects on perceived stress, burnout, insomnia, anxiety, depression, resilience, and well-being (10). Poor methodological quality was noted. A review of guidelines for reducing the mental health burden in healthcare workers found that 33 out of 41 articles recommended self-care: 50% of these suggested training for resilience building and stress management (11). Implementation strategies were lacking.

When BBMIC began at RISE NI, employees had already endured 10 months of severe COVID pandemic-related stress. Many felt overloaded and exhausted from daily job duties, home schooling children, and/or caring for relatives. In mass disasters, health workers carry the cumulative stresses of providing services while coping with the disaster’s effects on their own families (12). BBMIC was chosen by RISE because it is an evidence-based program that rapidly relieves symptoms of stress, anxiety, and trauma for health workers, children, families, and groups. With practice BBM can improve emotion self-regulation, social engagement, mental clarity, energy, and physical health (13–15). Management wanted to integrate BBM practices throughout RISE as ongoing institutional support for staff resilience and well-being.

Co-regulation of psychophysiological (mind-body) states by children and their caregivers is essential for healthy autonomic function, stress resilience, emotion self-regulation, relationships, and learning (4, 15–17). When adults interact with children, they communicate their own emotional states nonverbally. Children who have experienced excess stress, trauma, neglect, or disability tend to react more intensely and have more difficulty restoring emotional balance (9, 18). Educational settings provide opportunities for staff to reduce adverse effects of stress, trauma, and disabilities on children through positive co-regulation using simple, breathing exercises that support emotion self-regulation. Evidence supports the following mechanisms hypothesized to underlie the effects of BBM on psychophysiological states:

1. Changing the pattern of breathing changes afferent interoceptive messages from the respiratory system to central regulatory areas, including the limbic system, hypothalamus, thalamus, and insular, prefrontal, and anterior cingulate cortices (14, 16, 17, 19).
2. Slow breathing, particularly Coherent Breathing, balances the autonomic nervous system by reducing overactivity of the sympathetic nervous system (SNS) and boosting underactivity of the parasympathetic nervous system (PNS) (13).
3. Reducing SNS overactivity reduces energy expenditure. Increasing PNS activity restores energy reserves.
4. Breathing entrains the electrical activity of the brain. Voluntarily controlling breathing increases entrainment in critical areas such as insular cortex and amygdala (20, 21).
5. Slow breathing, such as Coherent Breathing, activates the social engagement system and enhances feelings of safety, trust, empathy, and connection (22–24).
6. Activating the vagus nerves by slow breathing may increases oxytocin release (4, 16).

Both RISE NI and the Breath-Body-Mind Foundation, a not-for-profit 501(c)3, evaluate programs and identify ways to improve outcomes. The objectives of this study were to:

1. Evaluate the effects of the Breath-Body-Mind Introductory Course on COVID-related stress among employees of RISE NI;
2. Reduce the risk of adverse effects from COVID-related stress;
3. Evaluate the effects of BBMIC on indicators of psychophysiological states and the consistency with hypothesized underlying mechanisms of action.

## Materials and methods

### Approval

RISE NI regional management approved the BBM training and service evaluation. Each manager obtained approval from their respective trusts. They determined that BBM Programs and Evaluations were part of service evaluation/service development, i.e., they did not meet criteria for research as defined by the National Health Services (NHS) UK Policy Framework for Health and Social Care Research, based on the Research Ethics Service. Consequently, BBM Programs and Evaluations did not require ethical approval from NHS Health Research Authority or Research Ethics Committee (25). Nevertheless, BBM provided RISE management with their informed consent form (see Supplementary Appendix A) which they approved.

### Recruitment

An email about BBMIC was sent to the RISE coordinators who advised their teams of the training. From among staff who volunteered to participate, team managers chose participants representing each discipline and forwarded the names to the BHSCT manager. The intent was for BBMIC training to maintain the transdisciplinary nature of the service. Initially, 40 places were allocated: 7 for each of 4 HSCTs and 12 for BHSCT. Unfilled places were offered first to the 4 HSCTs and last to BHSCT. The final allocation was: BHSCT 23; South Eastern HSCT 4; Southern HSCT 4; Northern HSCT 3; Western HSCT 5.

### Informed consent

Consent to participate in the program, test measures, and for publication, was obtained the week prior to BBMIC (see Supplementary Appendix A Consent Form). Team managers explained to staff the Informed Consent form, risks and benefits, the evaluation, confidentiality, the right to withdraw, and the potential for further BBM Training. Staff had an opportunity to have their questions answered. Staff registration to participate was considered consent.

### Participants

The participants, 39 adult women between ages 23 and 55 years, professional staff of the five HSCTs of RISE NI, included: speech and language therapists, occupational therapists, physiotherapists, clinical psychologists, behavior therapists, and therapy assistants. One male registered to participate, but he withdrew before BBMIC leaving an all-women group.

### Data collection and analysis

**Figure 1:**
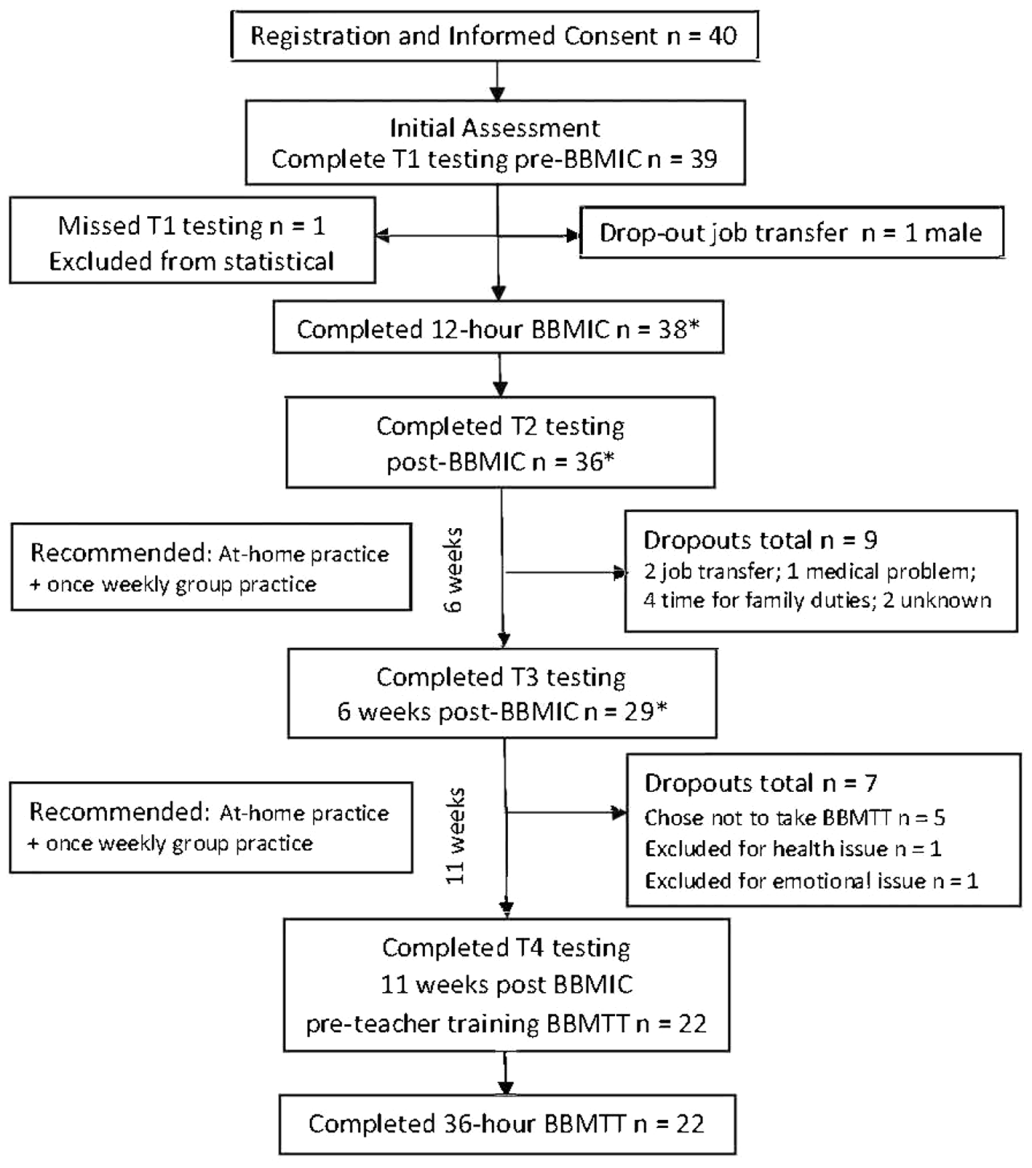
Starting with 40 registrants, the study flow diagram tracks participants through BBMIC and 11 weeks of follow-up and test measures while accounting for dropouts. * Indicates that the actual number of participants who completed BBMIC was 38. However, 36 completed T2 testing and 29 completed T3 testing. Because one person missed T1 testing, their scores were not included in the statistical analysis.

Data was collected between December 16, 2020 and April 14, 2021. Dependent measures were taken at four time points, pre-BBMIC training (T1), post-3 days of BBMIC (T2), post 6 weeks of solo and group practice (T3), and 11 weeks post training (T4). After BBMIC, some graduates (*n* = 22) participated in BBM Teacher Training Level-1, yielding additional data at T4 (see Figure 1).

Test scores were obtained online using questionnaires generated and collected through Surveymonkey.com. To maintain confidentiality and test security, each participant was assigned a unique Identification Code (ID) emailed to them individually by the BBM research team. RISE NI administrators and sponsors were blind to the match between IDs and individual participants and could not access their responses. To protect employee privacy, all tests were coded (no names or other identifying information appeared on test documents). The Master Code List was kept by Dr. Gerbarg, who was not affiliated with RISE, on a separate, secured memory stick. Since the study as a “low stakes” personal employee development opportunity, we did not anticipate any response tampering, multiple submissions or substitutions. The rater and data analyst were blind to participant IDs.

The RISE NI training contract did not allow time or funding to train more than one group, hence, the single group design. Comparison groups and randomized were not possible. Participants were a convenience sample of supervisor-nominated employees who volunteered to participate without additional pay beyond their usual salaries. BBMIC was taught during normal work hours.

The data collection plan was informed by the following assumptions and intentions:

1. Given the nature of their work and work-related changes necessitated by the COVID-19 pandemic, this sample of RISE NI staff would have higher-than-normal psychological stress.
2. On average, participation in BBMIC would be associated with measurable, meaningful, statistically significant improvements in perceived stress, engagement, revitalization, tranquility, physical exhaustion, and stress overload.
3. On average, participation in BBMIC would be associated with measurable improvements in indicators of positive change in psychophysiological states, such as tension, calmness, mood, sleep, energy, mental clarity and focus, friendliness, empathy, and body pain.
4. Having experienced personal relief and emotional healing, some participants would volunteer for a train-the-trainer program to learn how to incorporate BBM practices into their work.
5. The data would provide a formative evaluation of BBMIC to use to improve the training.

The data analysis plan was to measure baseline participant stress prior to BBMIC. Using the dependent measures we applied a General Linear Model (GLM) matched-pair, within-subjects, repeated measures design, a standard analytic approach, to test the level of change in each participant (26). Three conditions were set up for the factor “Time”: pretest (T1); post-3 days of BBMIC (T2); and after 6 weeks of solo and group practice (T3). SPSS uses Mauchly’s test for sphericity. The Bonferroni method was used for *post hoc* analysis.

Practice logs were analyzed to understand the degree to which the amount of group practice and at-home practice might correlate with changes in dependent measures. As in many studies of busy professionals, a drop-off in practice was anticipated. The pattern of those who left the program was analyzed using a *t*-test comparison of leavers vs. stayers to better understand potential biases in the overall result. In addition, we collected a matched set of measurements for those registered for Teacher Training Level 1 (TTL-1). The pre-test for TTL-1 served as an 11 weeks extension of post-time to measure persistence of effects. Effect size of changes were measured in the means of the key to matched sets of dependent measures calculating Cohen’s d.

We summarized the Program Evaluation questionnaire responses which assessed participants’ satisfaction with the training and their suggestions for improving BBMIC.

### Test measures

The *Perceived Stress Scale (PSS)* is a widely used, validated measure of perceived stress with reduced stress indicated by a lower score (27). Participants rate how often they have experienced stress-related symptoms over a period of at least 1 month on 10 items on a scale from 0 (never) to 4 (very often). Internal reliability has been estimated at *α* =0.78 (28). In addition, Cohen et al. report evidence of concurrent and predictive validity showing the results of various samples where the PSS correlated significantly with the Number of [stressful] Life Events, Impact of [stressful] Life Events, depressive symptomatology, and Health Center Utilization (27). PSS scores in normative samples have been published (28).

The *Exercise Induced Feeling Inventory* (*EFI*) has been shown to be sensitive to mood changes associated with yoga interventions (29, 30). Participants are asked to describe “how you feel at this moment in time” on a scale from 0 (do not feel) to 4 (feel very strongly). The EFI consists of 12 items grouped into 4 subscales: Positive Engagement, Revitalization, Tranquility, and Physical Exhaustion. Improvement in the first three subscales is indicated by a higher score and for the last subscale by a lower score. Estimates for internal reliability of EFI subscales range from *α* = 0.74 to *α* = 0.91 (29, p. 415). The subscales demonstrate good concurrent and discriminant validity with existing measures of mood and affect (29, p. 417). The data also suggest that EFI is highly sensitive to changes in feeling states that occur with exercise (29, p. 419).

The *Stress Overload Scale-Short (SOS-S)* is comprised of 30 items designed to measure “stress overload,” a state described in stress theories as occurring when demands overwhelm resources (31). A 5-point Likert scale (1 = not at all, 5 = a lot) indicates subjective feelings and thoughts experienced over the prior week. Two factors underly stress overload: Personal Vulnerability (PV) and Event Load (EL), measured by two distinct but correlated subscales. Higher total scores indicate higher levels of stress overload. SOS-S internal consistency is excellent (Cronbach’s alphas > 0.94 for both subscales and the measure as a whole). Test–retest reliability is good (coefficients averaging 0.75 over 1 week). Significant correlations with other measures of stress and illness demonstrate construct validity. Criterion validity has been shown in prediction of illness and abnormal cortisol responses following a stressful event (32). Amirkhan also suggests a “categorical scoring option” for separating participants into risk categories using norm sample group means as dividing points on the Personal Vulnerability (μPV =  9.15) and Event Load (μEL = 12.15) subscales. Those scoring in the High EL-High PV category were found to be at highest risk for developing a stress-related health condition.

*Indicators of Psychophysiological State* is a subjective non-validated self-assessment of 22 items, created by authors Gerbarg and Brown to document changes in psychophysiological state that reflect stress responses, emotional state, and perceptions of oneself, other people, and the environment, as delineated in the Polyvagal Theory of Stephen Porges and discussed below (15, 16, 33). Items are based on awareness of physical sensations (*interoception*), energy, emotions, cognitive functions, attention, and aspects of social engagement, including awareness of others, attitude towards others, connectedness, and empathy. Some items are similar to those in the Body Perception Questionnaire Short Form, a validate measure develop by Porges (34, 35).

*Compliance* with the recommended practice time was assessed using home Practice Logs submitted by participants and attendance records kept by BBM teachers during weekly group practice sessions.

*Qualitative Data*: Following BBMIC, participants were asked three open-ended questions at T2: (1) What did you like the most about BBMIC?, (2) What can be improved in the BBMIC?, and (3) How can BBM practices be helpful to students?

### Intervention: Breath-Body-Mind Introductory Course

BBMIC includes a 12 h manualized training provided live online 4 h/day for 3 consecutive days followed by 6 weeks of once-a-week online group practice (45 min per session) and daily home practice (recommended 20 min per day of coherent breathing with some movement practices). This course teaches participants how to become more aware of their own psychophysiological states and how to use BBM techniques to balance their own stress response systems (sympatho-vagal balance). By developing self-awareness and regulation of their own psychophysiological state, participants become better able to co-regulate the emotional states of others. In collaboration with Jyoti Manuel,^1^ methods for working with children with special needs were incorporated into the BBMIC.

In BBM programs, Coherent Breathing, the foundational practice, is used alone and with attentional focus, synchronized movements, visualization during Breath Moving, music, and audio track or voice pacing. To optimize relaxation, participants are encouraged to be in a comfortable, supported position and to exert as little effort as possible during Coherent Breathing paced at 5–6 cpm (cycles per minute). This gentle cyclical breathing (without breath pauses or holds) has calming effects, reduces SNS activity, increases PNS activity, lowers blood pressure, and induces synchronized alpha waves across large areas of cerebral cortex (17, 19, 20, 36–39). In the resulting psychophysiological state, the individual feels both calm and alert.

Each day had 3 Rounds that included: activating practices, such as tapping the body to music or “Ha” breath (no more than 1 or 2 min); autonomic balancing practices that coordinate breathing with movement, for example, breathing at 5 cpm while making synchronized arm circles; two deep relaxing sighs, a brief top-down muscle relaxation; Coherent Breathing (or resonant breathing) paced at 5 cpm, starting with 7 min and working up to 20 min; and Breath Moving (imagining the movement of breath inside the body in a sequence of circuits) (See Supplementary Appendix B – Table B1 Schedule and Table B2 Description of Practices). This can be followed by a bottom-up body scan, soft relaxing music, or Open Focus Attention Training (40, 41). The practice finishes with a few minutes of rest, lying down if possible.

## Results

### Participants

After baseline tests were obtained from 39 RISE NI employees, the only male in the group dropped out. Subsequently, 38 female employees (administrators and front-line workers) from the five district offices of RISE NI participated in BBMIC. Their ages ranged from 23 to 55 years. Among the 38 participants included in the statistical analysis were: 8 behavioral specialists, 8 occupational therapists, 7 speech and language therapists, 5 physiotherapists, 7 therapy assistants, 2 social workers, and 1 clinical psychologist. These included 8 Team Leaders. Figure 1 tracks the number of participants who completed tests at each time point and the reasons for study dropouts.

### Assessment measures: results and analysis

#### Perceived stress scale

Using Welch Modified Two Sample *t*-Test in R-Studio (Version 1.2.5019 BSDA library), showed that at baseline (T1), PSS mean score of the 38 women participants (PSS = 18.3 ± 6.108 SD) was significantly higher (*p* < 0.0001) than the estimated population mean in a PSS norming sample of 1,406 women (μ = 13.7 ± 6.6 SD) (28). Participants who chose to participate in BBM Teacher Training Level-1 (*n* = 21) completed the PSS 11 weeks post BBMIC during the week before teacher training. A matched pair *t*-test comparing the PSS BBMIC baseline (T1) mean of 18.19 with the pre-BBMTTL1 (T4) mean of 15.67 was statistically significant (*p* < 0.046) with an effect size estimate using Cohen’s *d* = 0.46 in the moderate range.

### Impact of the BBMIC training on dependent measures

#### Stress overload scale-short

**Figure 2:**
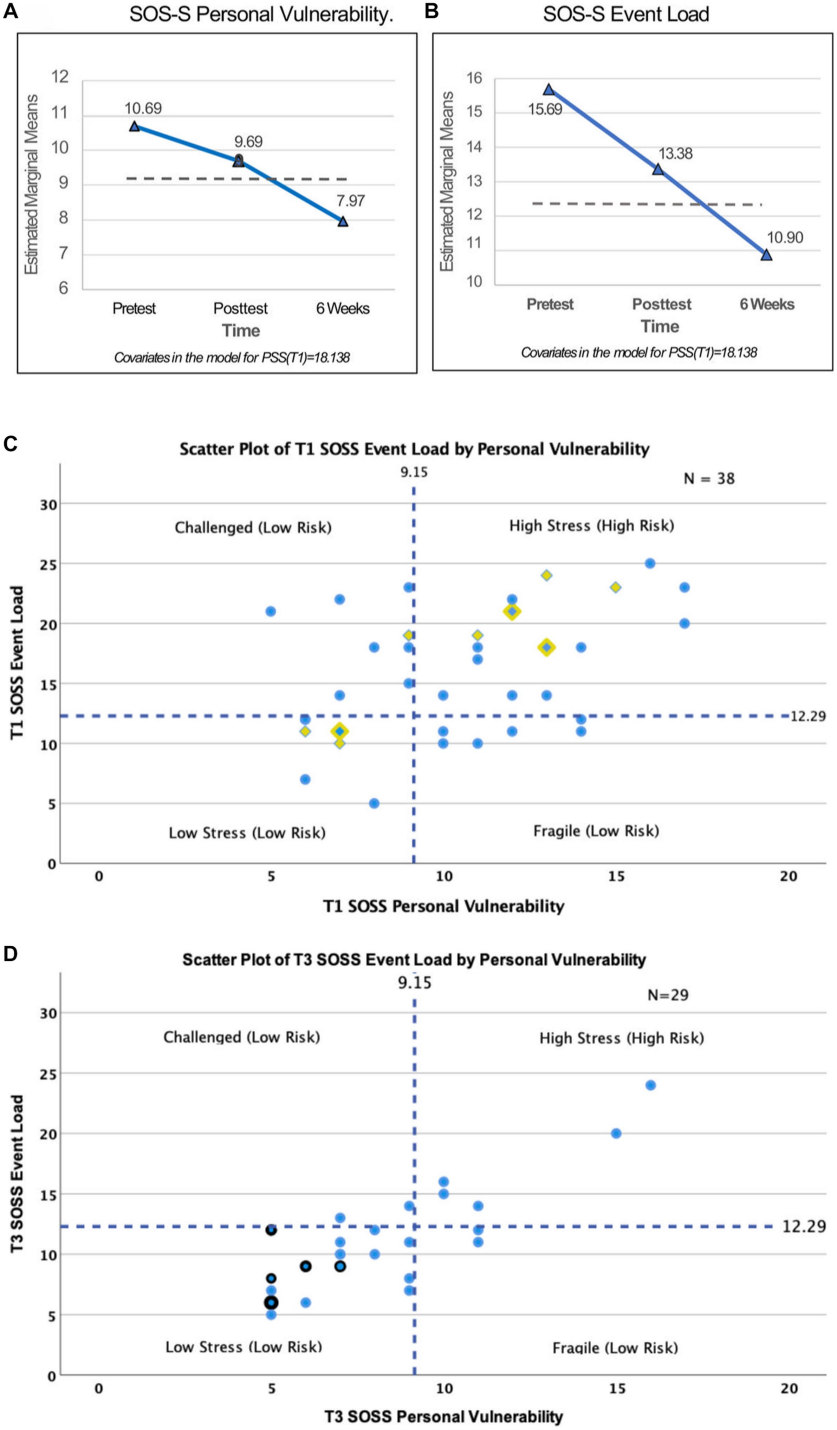
Stress Overload Scale-Short (SOS-S). **(A)** Pretest (T1) estimated marginal means of the Personal Vulnerability (PV) subscale was 10.69 and decreased to 9.69 at T2. Six weeks later (T3) PV score decreased significantly to 7.97 (*p* < 0.001), below the normative mean score (dashed line). The T1 Perceived Stress Scale (PSS) total score was used as a covariate, value = 18.138. **(B)** Pretest (T1) estimated marginal means of the Event Load (EL) subscale was 15.69 and decreased significantly to 13.38 (*p* < 0.028) at T2. SOS-S estimated marginal means decreased significantly to 10.90 (*p* < 0.001) at T3 and was below the normative mean score (dashed line). The T1 PSS total score was used as a covariate, value = 18.138. **(C)** A scatterplot of Baseline (T1) scores shows Event Load on the vertical axis and Personal Vulnerability on the horizonal axis. Dashed lines represent the normative population mean scores (μ_PV_ = 9.15) and (μ_EL_ = 12.29). The resulting four quadrants indicate characterizations as Challenged (Low Risk), Low Stress (Low Risk), Fragile (Low Risk) and High Stress (High Risk). Blue circle = 1 subject who stayed in the study; Yellow diamond = 1 subject who dropped out anytime between T1 and T4; Blue dot inside yellow diamond = 2 participants with identical scores, one who stayed in the study and one who dropped out. **(D)** 6 weeks post (T3) matrix with Event Load on the vertical axis and Personal Vulnerability on the horizonal axis. Dashed lines are at the normative population mean scores (μ_PV_ = 9.15) and (μ_EL_ = 12.29). Blue circle = 1 subject; Blue circle with black border = 2 subjects with the same score.

The pretest (T1) estimated marginal means of the *Personal Vulnerability* (PV) subscale of the SOS-S was 10.69 (*n* = 38) and decreased significantly to 9.69 after BBMIC. When subjected to a Multivariate Repeated Measures analysis of Covariance, the dependent variables yielded an *F* = 8.668 with df = 12 and *p* < 0.001. Pairwise Comparisons of the marginal means across the 3 time periods for SOS-S Personal Vulnerability (*n* = 29) (see Figure 2A) shows the initial decrease from the T1 mean of 10.69 to the T2 mean of 9.69 in not statistically significant (*p* < 0.287), but the T1 mean compared to the T3 mean of 7.97 is significant (*p* < 0.001). The T1 PSS total score was used as a covariate with a value of 18.138. In a similar analysis of the SOS-S *Event Load* subscale the Pairwise Comparisons of the marginal means (*n* = 38) (see Figure 2B) shows a significant drop from 15.69 (T1) to 13.38 (T2) (*p* < 0.028). The decrease in marginal means from 15.69 (T1) to 10.90 (T3) is also statistically significant (*p* < 0.001).

The SOS-S can be analyzed by placing each participant’s scores on a 2 × 2 matrix (Figure 2C) with Event Load as the vertical axis and Personal Vulnerability on the horizontal. The graph divides into 4 categories by a vertical line drawn at the Personal Vulnerability population norm (μ_PV_ = 9.15) and a horizontal line drawn at the Event Load population norm (μ_EL_ = 12.29). The resulting quadrants sort participants into “Challenged,” “High-Stress,” “Low- Stress” or “Fragile” categories. The pretest (T1) SOS-S identified 17 of the 38 participants (45%) in the High Stress category (32). In Figure 2C data points for participants who later dropped out of the study “Leavers” are designated by yellow triangles (*n* = 9). Those who stayed appear as blue circles (*n* = 29). A blue circle over a yellow triangle indicates a score (data point) that is the same for one Stayer and one Leaver. Figure 2D shows SOS-S at T3 (*n* = 29) when only 5 of the 29 (17%) were identified as “High Stress.” Thus, the percentage of participants at high risk for developing a stress-related disorder at T1 decreased substantially from 45 to 17% at T3.

#### Exercise induced feelings inventory

**Figure 3:**
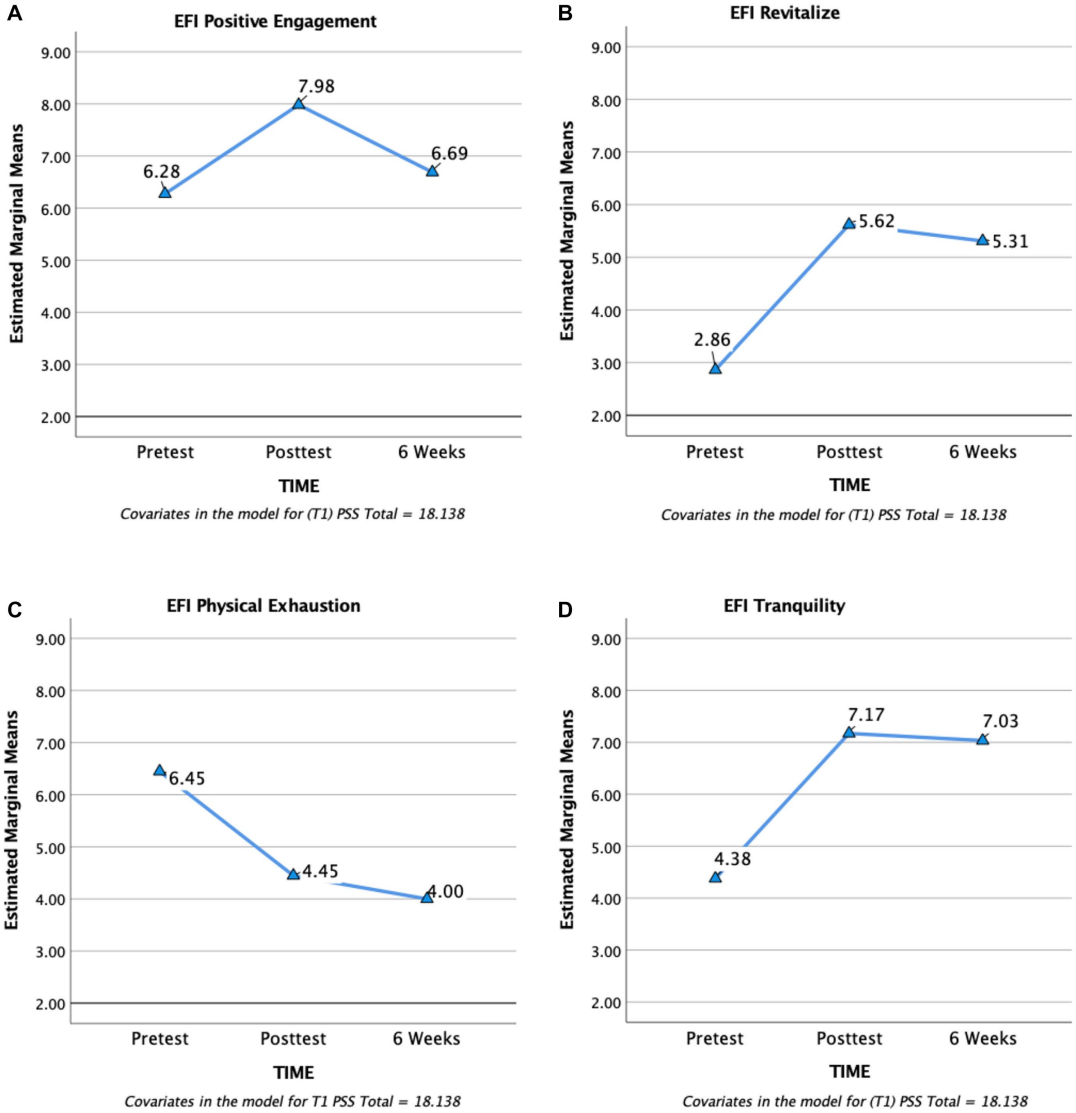
EFI Subscales Estimated Marginal Means over time. **(A)** Positive Engagement. **(B)** Revitalization. **(C)** Physical Exhaustion. **(D)** Tranquility. The three time points are pretest baseline (T1), 3 days posttest (T2), and 6 week posttest (T3). The initial improvement in engagement from T1 to T2 was not sustained at T3. However, statistically significant improvements from T1 to T2 in revitalization, physical exhaustion, and tranquility were maintained at T3.

Pairwise comparisons of marginal means for the EFI subscales (*n* = 29) are shown in Figure 3:

3A. EFI Positive Engagement means showed statistically significant increases from 6.27 (T1) to 7.98 (T2) (*p* < 0.001), but dropped to to 6.69 (T3) which was still significantly better that at T1 (*p* < 0.037).
3B. EFI Revitalization Scale means showed a statistically significant increase from 2.86 (T1) to 5.62 (T2) (*p* < 0.001), but dropped slightly to 5.31 (T3), which was still significantly better than at T1 (*p* < 0.001). The difference of 0.31 between T2 and T3 is not significant (*p* < 1.0).
3C. EFI Exhaustion Scale means showed statistically significantly decreases from 6.45 (T1) to 4.45 (T2) (*p* < 0.018) and 4.0 (T3) (*p* < 0.014).
3D. EFI Tranquility Scale means showed statistically significant increase from 4.38 (T1) to 7.27 (T2) (*p* < 0.001) and stayed relatively unchanged to 7.03 (T3) (*p*_T2−T3_ < 1.0).

#### Subjective changes in indicators of psychophysiological state

**Figure 4:**
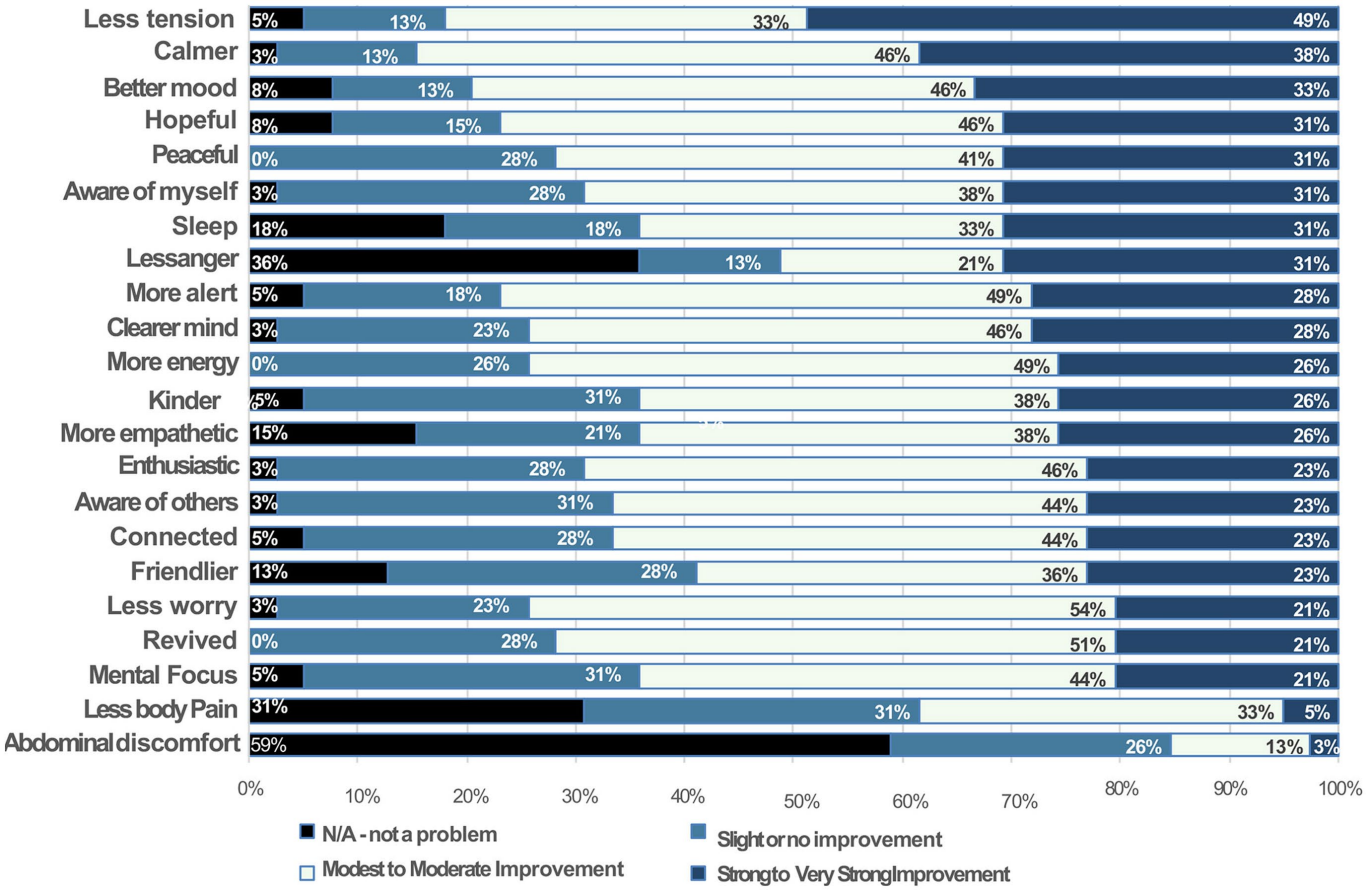
Subjective Changes in Indicators of Psychophysiological State from Baseline (T1) to 3 days posttest (T2): The vertical axis lists psychological and physical items, including qualities, functions, and states that reflect or are strongly associated with psychophysiological states. Each bar shows the proportion (%) of participants who rated each item on a scale of severity condensed into four categories from left to right: “not a problem,” “slight improvement,” “modest to moderate improvement,” or “strong to very strong improvement.”

Participants rated the level of improvement they experienced after the first 3 days of BBMIC (T2) on a 7-point Likert-type scale, which was collapsed into five categories by combining “strong” with “very strong improvement” scores, as well as combining “modest” with “moderate improvement” (see Figure 4). No subjects reported “no improvement.” Therefore, that category was dropped, leaving four categories. On average, including all items, approximately 10% of respondents felt that an item was not a problem for them; about 22% reported slight improvement; 41% modest to moderate improvement; and 28% strong to very strong improvement in 22 indicators of psychophysiological states (*n* = 36). For example, calmness and peacefulness are associated with feelings of safety and higher PNS activity versus tension, worry, and anger, which are associated with feeling threatened and defensive states of higher SNS system activity (15, 16, 19).

#### Compliance

All participants who completed BBMIC were asked to keep a log of the time they spent each day practicing BBM techniques on their own (Solo) and in Group Practice Sessions led by BBM senior instructors throughout the 6 weeks period of practice. The goal for 100% compliance was 20 min/day solo practice (20 min/day × 6 days/wk. × 6 wks = 720 min) plus one 45 min group practice session per week (45 min/wk. × 6 wks = 270 min). Of the 38 participants, 34 submitted practice logs weekly through SurveyMonkey (see Table 1). Based on the mean practice times reported, participants met 85% of the goal for Group Practice; 44% of the goal for solo practice; and overall 55% of the total practice goal.

**Table 1:** Group mean practice times and compliance with practice goals.

	Mean practice time over 6 weeks *n* = 34		
	Mean group Practice minutes	Mean solo Practice minutes	Mean total Practice minutes
Practice goal	270	720	990
Reported practice	230.3	316.4	546.7
Mean % of practice goal completed	85%	44%	55%

During the 6 weeks of practice from T2 to T3, participants reported their group practice time and solo practice time, which were combined for total practice time in minutes. The group mean reported practice time compared to group practice goals yielded the compliance as the percent (%) of practice goal completed.

#### Qualitative data

**Figure 5:**
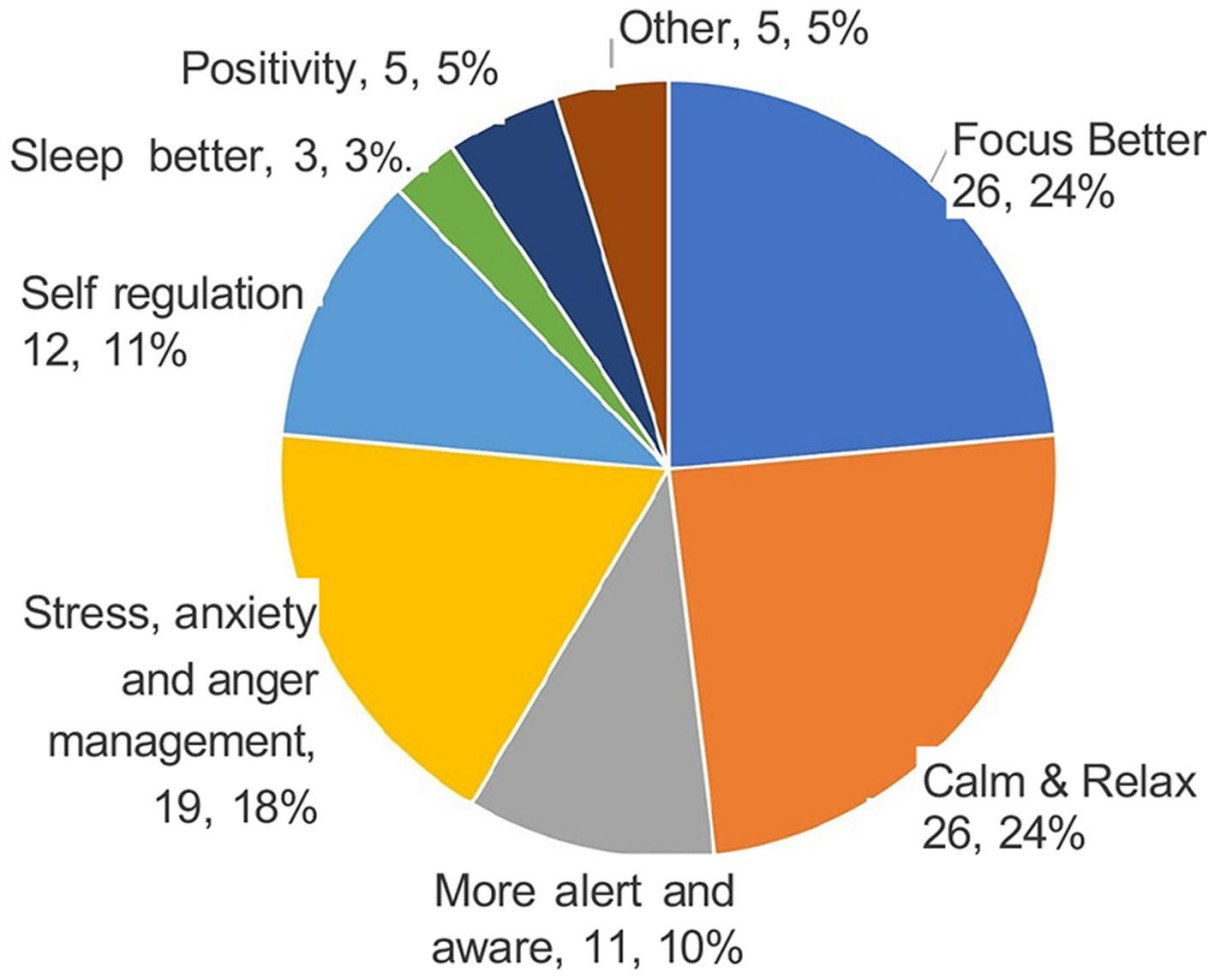
Program Evaluation at week 6 (T3): How Can BBM Practices be Helpful to Students? Each sector of the pie represents a way in which the participants thought that students could benefit from BBM practices. The numbers show how many participants made a comment related to each sector and the percentage (%) of the total comments they represented.

A total of 106 comments submitted by 34 of the participants were organized into eight themes for each question. The most frequent responses were as follow:

1. What did you like the most about BBMIC? Breathing techniques 25%; scientific background and examples 11%; practicality, applicability, and easiness of techniques 11%; environment/organization of the course 9%; time for self- care 7%; tapping 7%; other.
2. What can be improved in the BBMIC? Move lectures to day-1 19%; more breakout rooms 14%; smaller work groups 8%; in-person workshops 8%; other.
3. How can BBM practices be helpful to students (see Figure 5). Better focus 24%; calm and relax 24%; stress/anxiety and anger management 18%; self-regulation 11%; more alert and aware 10%; positivity 5%; other.

### Adverse reactions

Participants were encouraged to report any adverse experiences during BBMIC group sessions and home practice. During each session the BBM teachers did “check-ins” by asking the participants how they were feeling and encouraging them to share positive and negative experiences related to the practices. In addition, visually monitoring students enabled teachers to observe how they were doing the breath practices and any signs of tension, distress, or discomfort.

No serious or persistent adverse reactions occurred. On the first day of learning Coherent Breathing, several participants reported that they felt uncomfortable as though they could not prolong their breath enough. However, by the second day, all but a few were breathing more comfortably at 5 cpm. Some experienced sad feelings transiently. Several participants had recently lost close relatives or friends and were in grief. They took time out as needed. One woman could not reduce her respiratory rate below 20 cpm. She was referred for medical evaluation. A few participants noted transient muscle aches as tension was released. Most reported feeling better physically and emotionally by the end of each BBMIC session. One participant with asthma and allergies found it stressful to slow her respiratory rate to 5 cpm. Instead, she was given an audio track to pace her breath at 6 cpm which was more comfortable. Most people find that over time Coherent Breathing improves asthma, but when they are having acute asthma symptoms, they may not be able to breathe at 5 cpm and trying to do so may exacerbate their difficulties. This can be prevented by instructing them to use Breath Moving during Coherent Breathing and, if necessary, by breathing at a slightly faster rate.

Individuals recovering from COVID are instructed to do coherent breathing lying in prone position and to breathe very gently at a rate that does not cause any strain. They are also taught to do the other breath practices very gently and rest when needed. One pregnant participant was uncomfortable sitting. She was instructed to lie down during Coherent Breathing. She was advised not to do breath holds, forceful breaths, or rapid breathing.

## Discussion

### Background and context

#### COVID effects on the regional integrated support for education Northern Ireland

Healthcare agencies, like RISE NI, have been subjected to increased levels of psychological stress since the COVID-19 pandemic began (12, 42). As conscientious caregivers, they felt responsible not only for the wellbeing of the children and families they serve, but also for their own families. Moral distress, a precursor to moral injury, can occur when staff cannot provide the needed services, for example, during the COVID pandemic (43). Evidence suggests that in agencies providing services for children with disability or developmental delay, the four main factors that impact the effectiveness of workers in providing services are: engagement with the workplace, engagement with clients, professional capacity, and staff wellbeing (44).

#### The effects of COVID on children and families in Northern Ireland

A study led by the National Children’s Bureau highlighted the impact of the COVID pandemic on children and youth with Special Educational Needs and Disabilities (SEND), their families, and those who support them (18). The report noted: (1) fear, insecurity, and worry about the health of their children and themselves, (2) social isolation and loneliness, already problematic for children with SEND, was exacerbated by the closing of schools and other activities as well as quarantine requirements, (3) loss of support from caregivers and support workers from other agencies who could no longer do home visits, (4) loss of learning and development during school lockdowns or changes in how schools functioned, and (5) worsening of stress on the already strained services.

Working under such adversity affects stress response systems and leads to psychophysiological states of defense, as evidenced by the baseline data which showed high levels of perceived stress, stress overload, and physical exhaustion, as well as low levels of tranquility, energy, mood, and social engagement. Many of these manifestations of the stress-related psychophysiological states are associated with impaired effectiveness at work and at home, poorer quality of life, ill health and increased vulnerability to stress-related disorders.

#### Neurophysiological theories: the Polyvagal theory and the Vagal-GABA theory

According to the Polyvagal Theory of Stephen Porges, we can perceive the environment as safe (“green zone”), unsafe (“orange zone”), or life threatening (“red zone”). When we feel safe, the myelinated fibers of the parasympathetic nervous system (PNS) (within the vagus nerves) are orchestrating an autonomically balanced psychophysiological state of elevated heart rate variability (an indicator of health and longevity), social engagement and non-defensiveness, wherein our emotions are well regulated, and we are able to trust, bond, love, be intimate, self-soothe, heal, feel empathy and compassion, and cooperate with others (15, 16, 45).

When we feel threatened (unsafe), the sympathetic nervous system (SNS) becomes more dominant, inducing a psychophysiological state of defense in which heart rate variability is low, social engagement declines, and we become mobilized for fight or flight. This behavioral activation is necessary for survival, but is accompanied by emotion dysregulation, anger, fear, mistrust, hypervigilance, and overreactivity (4, 16). In a situation perceived as life-threatening, when we can neither fight nor escape, the nervous system may default to the evolutionarily older unmyelinated vagal pathways associated with a state of low heart rate variability wherein the social engagement network cannot function. In addition, this can lead to freeze reactions, disconnection, dissociation, or numbing.

The neurophysiological theory of mechanisms contributing to the effects of voluntarily regulated breathing exercises articulated by Brown and Gerbarg (4, 14, 39) hypothesizes that:

1. Changing the pattern of breathing changes afferent interoceptive messages from the respiratory system (mechanoreceptors, chemoreceptors, and baroreceptors) that ascend through the vagus nerves to brainstem nuclei and from there to the main central regulatory areas, including the limbic system, hypothalamus, thalamus, interoceptive (insular) cortex, prefrontal cortex, and anterior cingulate cortex (16, 17, 46–49).
2. Slow breath exercises, particularly Coherent Breathing, balances the autonomic nervous system by reducing the overactivity of the sympathetic branch, as occurs in anxiety disorders and PTSD, and by boosting the underactivity of the parasympathetic branch (13, 38, 50, 51). This is consistent with the changes perceived stress and psychophysiological state.
3. When the sympathetic nervous system is over-active it consumes more energy and generates more free radicals as byproducts. Reducing sympathetic system overactivity reduces this excess energy expenditure. The parasympathetic system is responsible for restoring energy reserves. Increasing parasympathetic system activity restores the depleted energy reserves. The net result is increased energy and decreased exhaustion. Participant reporting of significant improvements in exhaustion at T2 and T3, despite the fact that their workloads and stressors were virtually the same at all test points are consistent with hypothesis #3.
4. The Vagal-GABA Theory of Inhibition hypothesizes that slow coherent breathing increases levels of gamma-aminobutyric acid (GABA), the main inhibitory neurotransmitter in the brain. Furthermore, increased GABA transmission from the prefrontal cortex and insular cortex could inhibit the overactivity that occurs in the amygdala in anxiety disorders and PTSD (52). A Mass Resonance Spectroscopy (MRS) study showed increased levels of GABA in the thalamus of patients with Major Depression who participated in a 12 week program of yoga and Coherent Breathing at 5 cpm (52). Test results showing improvements in tension, worry, calmness, mood, peacefulness, sleep, and tranquility are consistent with improved emotion regulation and inhibition of amygdalar over-reactivity.
5. Breathing entrains the electrical activity of the brain. Voluntarily controlling the breath pattern further increases the entrainment in critical areas such as the insular cortex and amygdala (20, 21). Slow breathing induces synchronous alpha waves across broad areas of the cerebral cortex, consistent with a state of calm attention and awareness, as indicated on the Subjective Indicators of Psychophysiological States.
6. Slow breathing, such as Coherent Breathing, activates the social engagement system and enhances feelings of safety, trust, friendliness, empathy, connection, and bonding (4, 22–24), as documented by the Subjective Indicators of Psychophysiological States.
7. Slow gentle breath practices may increase levels of oxytocin, enhancing feelings of closeness, trust, safety, bonding, and love (4, 16, 53) that are consistent with items on the Subjective Indicators of Psychophysiological States.

Breath-Body-Mind practices may improve both stress resilience and trauma recovery through: (1) strengthening and activating the myelinated pathways of the PNS such that the individual develops greater ability to sustain the feeling of safety and calmness, even under multiple prolonged stressors and (2) learning to activate the PNS while reducing overactivity of the SNS, providing a means to calm down and shift out of the perceived threat “orange zone” and back into the feeling of safety “green zone” (15, 16, 19).

#### Breath-Body-Mind affects psychophysiological states during and after mass disasters

In mass disasters and in clinical studies, BBM practices have significantly reduced symptoms of anxiety disorders, stress, depression, and post-traumatic stress disorder, including in populations affected by mass disasters, for example, the 2001 New York World Trade Center attacks; Gulf Horizon oil spill; military service; war and genocide in South Sudan, Rwanda, Myanmar, and Ukraine; kidnapping, and trafficking in Nigeria and South Sudan (54–57). BBM programs are specifically designed for situations in which a small number of caregivers with a limited amount of time must serve a large population of individuals experiencing stress, trauma, disaster, or illness such as COVID-19. Traditional mind-body programs offer hundreds of worthwhile practices, but these may require months or years of training. In contrast BBM has distilled sets of simple, relatively short practices that are safe and effective for most people, can be easily modified for those with physical or mental conditions, are accepted across cultures and ethnicities, require no equipment or supplies, and can be delivered by community extenders.

In 2002, Jyoti Manuel founded Special Yoga, a program for children with special needs, disabilities, developmental disorders, and/or trauma. She has worked for the National Health Service (NHS) and education authorities, providing in-house training and programs in schools, and through clinical teams of occupational therapists, physiotherapists, and other specialists. In 2019, Manuel began BBM training and eventually became a BBM Level-4 teacher. She found that the breath-centered practices were easily adapted and rapidly effective for the children she treats. Manuel, Gerbarg, and Brown integrated Special Yoga with BBM techniques for children with learning disabilities and other special needs and for the wider population of children and families affected by the COVID pandemic, stress, war, and trauma. Manuel contributed her knowledge and experience to the development of the child curriculum of BBM training and she co-taught RISE NI staff during the BBM courses.

The neurophysiological platforms affected by BBM techniques support restoration of autonomic balance (reduce the overactivity of the SNS and increase the underactivity of the PNS), the sense of safety, and interoception (perceptions of sensations arising from inside the body). Furthermore, evidence suggests that these changes activate the social engagement and Bonding systems and reduce defensive behaviors (15, 16, 45). Also, the breath and movement exercises can improve respiratory function, endurance, blood pressure, and inflammation (58, 59).

Participation in BBMIC was associated with significant improvements on PSS, SOS-S, EFI, and Subjective Indicators of Psychophysiological State. These changes were in the expected direction of positive improvement, based on previous studies. They are also consistent with a shift from a defensive state of increased sympathetic tone and decreased parasympathetic tone, described by Porges as the “Orange Zone” to a state of feeling safe (non-defensive) with increased parasympathetic tone and reduced sympathetic tone, the “Green Zone” (15, 16). Based on their personal experience of the changes that occurred during BBMIC, the RISE staff anticipated that the practices would benefit their students in comparable ways: calm and relax; better focus; stress/anxiety and anger management; self-regulation; and more alert and aware.

*This study met its three objectives:*

1. The study evaluated the effects of the BBMIC on COVID-related stress in RISE NI staff.
2. Participation in the BBMIC was associated with significant improvements on standardized measures of stress, PSS and SOS-S. The SOS-S matrix demonstrated substantial reduction in the number of participants who were at high risk for developing a stress-related condition, thereby reducing their risk of adverse effects from COVID-related stress;
3. The study evaluated and quantified the degree of subjective improvement on 22 indicators of psychophysiological states following BBMIC. The PSS, SOS-S, and EFI subscale items are also associated with psychophysiological states, adding evidence of improvement on standardized tests. The positive direction of all of these changes is consistent with the hypothesized underlying mechanisms of action.

### Study strengths

Study strengths included the use of manualized, previously tested BBM interventions taught by experienced faculty (Dr. Brown, Dr. Gerbarg, Jyoti Manuel, and BBM senior teachers). Another strength was the use of codes to preserve participant confidentiality (particularly in their workplace) and blinding of those who collected and analyzed the data. The faculty were trained and led by Dr. Brown and Dr. Gerbarg who created the BBM programs and who have taught BBM for over 15 years to disaster survivors and others with stress, trauma, and stress-related physical conditions. To provide high quality, consistent teaching, all BBM assistant faculty were trained and certified in BBM Levels 3 or 4 by Dr. Brown and Dr. Gerbarg. Additionally, thrice daily meetings enabled the teachers to discuss the participants and receive guidance and supervision from Drs. Brown and Gerbarg. Thus, problems could be addressed quickly. Individual coaching was provided as needed for participants who had difficulty performing the practices.

### Study limitations

This study of professional healthcare employees had no control group. A randomized controlled study is needed to compare the impact of BBMIC on employees with a similar group who did not participate in the intervention. One cannot dismiss the possible effects of time away from work duties, interaction with BBM faculty, and group interaction. It is also possible that environmental conditions improved, reducing the load of stressors. This is unlikely because the COVID pandemic did not abate during the study, nor was there relief from the workload or psychosocial pressures.

The selection process may have favored employees who were more motivated to learn mind-body practices. Future studies could explore whether participant motivation correlates with responses to BBMIC.

Because the participant group was small, larger studies are needed to validate and extend the findings; studies in other settings are needed to expand generalizability. The one male who signed up for the study dropped out before the intervention. Inclusion of a larger proportion of males would address the possibility of gender-related differences in response. Studies are also needed for more ethnically diverse populations.

All measures were subjective. The use of biological measures in future studies could provide objective evidence of changes in psychophysiological states, for example, measures of resting pulse, blood pressure, respiratory sinus arrhythmia or heart rate variability, cortisol levels, or inflammatory markers. Brain scan studies, including connectivity, would deepen our understanding of the neural mechanisms involved.

BBM is an interactive multi-component program that includes sequences of breathing, movement, Open Focus attention training, interoceptive awareness (akin to mindfulness), and group processes. Given that each exercise is chosen for its specific effects, one cannot clearly differentiate the relative contribution of the components to the overall effect.

### Future directions

This study evaluated the effects of BBMIC on professional staff only. The next step would be to study how the program affects the way staff perform their jobs and the effects on children and families receiving care. Studying the effects of improvements in staff self-regulation on the children and the role of co-regulation in the children’s responses would be worthwhile. The negative impact of COVID-related psycho-social stressors on children’s mental health, social–emotional development, and, particularly for those with special needs, those affected by trauma, and marginalized students, cannot be overstated.

Voluntarily controlled breath practices open a portal to interoceptive communication networks which upregulate or downregulate brain functions. Breath practices can be used as non-invasive probes to explore changes in connectivity and neurotransmitter levels, shedding light on neurophysiological events that underly the observed clinical responses (60). It is possible to prescribe specific breath practices that integrate easily into treatments for a wide range of mental and physical disorders, as well as for prevention and performance enhancement. The practices used for everyday stress can be used for mass disasters to support better functioning and recovery.

## Conclusion

Breath-Body-Mind programs have been shown to reduce symptoms of anxiety, depression, and PTSD in survivors of mass disasters internationally. The COVID pandemic caused acute and chronic psychological stress for healthcare workers, who experienced increased workloads while they and their families were suffering from COVID-related stressors. The stress was exacerbated by reductions in staff due to illness, family needs during school closures, worsening problems of children with disabilities and other special needs, and loss of other support services.

The staff at RISE NI showed elevated levels of stress on two standardized measures. Baseline data were consistent with states of defensiveness, fear, worry, and exhaustion. Completion of the Breath-Body-Mind Introductory Course (BBMIC) was associated with improvements in measures of stress, personal vulnerability, sense of work overload, revitalization, tranquility, physical exhaustion, mood, sleep, and indicators of positive, emotionally meaningful relatedness, including feelings of connectedness, kindness and empathy. These changes are consistent with the hypothesized psychophysiological shift from feeling unsafe (increased sympathetic activity) to feeling safe (increased parasympathetic activity). In accord with polyvagal theory, the state of feeling safe supports the social engagement system, including the ability to feel trust, close, connected, and empathic. These essential components of stress resilience are also necessary for optimal co-regulation and work performance during interactions with co-workers, children, and families, particularly during times of prolonged, increased stress, such as the COVID pandemic, war, population displacements, and other mass disasters.

Breath-centered mind-body programs, such as Breath-Body-Mind, may support staff recovery from COVID-related and other stressors. In effect, the BBMIC served to counteract adverse effects of COVID-related psychological stress on mental and physical health. Training healthcare employees in BBM may serve a preventive role by enhancing their abilities to tolerate stress and maintain their own wellbeing in a more robust psychophysiological state. Further research is needed to validate and extend these promising findings towards the development of breath-centered individual and group treatments.

## Data availability statement

The original contributions presented in the study are publicly available. This data can be found here: https://dataverse.harvard.edu/dataset.xhtml?persistentId=doi:10.7910/DVN/NO0VGP. 

## Ethics statement

Ethical review and approval was not required for the study on human participants in accordance with the local legislation and institutional requirements. Written informed consent for participation was not required for this study in accordance with the national legislation and the institutional requirements.

## Author contributions

All authors listed have made a substantial, direct, and intellectual contribution to the work and approved it for publication.

## Funding

Funding was provided through the Belfast Health and Social Care Trust (BHSCT) of Northern Ireland. BHSCT only paid for the Breath-Body-Mind teachers and technical staff to provide the BBMIC to its employees. PG, RB, and VC provided their time *pro bono* to do the evaluation and prepare the paper for publication.

## Conflict of interest

PG and RB have written books and book chapters about Complementary and Integrative Medicine, including breath-centered mind-body practices. They sometimes receive financial remuneration or honoraria for teaching mind-body practices. They are Co-Founders and members of the Board of Directors of the Breath-Body-Mind Foundation, a not-for-profit 501(c)3 that provides pro bono crisis relief programs for survivors of mass disasters, scholarships for applicants who cannot afford to pay for training, and grants for research projects.

The remaining authors declare that the research was conducted in the absence of any commercial or financial relationships that could be construed as a potential conflict of interest.

## Publisher’s note

All claims expressed in this article are solely those of the authors and do not necessarily represent those of their affiliated organizations, or those of the publisher, the editors and the reviewers. Any product that may be evaluated in this article, or claim that may be made by its manufacturer, is not guaranteed or endorsed by the publisher.

## Footnotes

^1^ https://www.specialyoga.co.uk

## Abbreviations

BBM: Breath-Body-Mind
BBMIC: Breath-Body-Mind Introductory Course
BBMTTL-1: Breath-Body-Mind Teacher Training Level-1
BHSCT: Belfast Health and Social Care Trust
cpm: cycles per minute
EFI: Exercise Induced Feelings Inventory
EV: Event Load
PNS: parasympathetic nervous system
PSS: Perceived Stress Scale
PV: Personal Vulnerability
RISE NI: Regional Integrated Support for Education, Northern Ireland
SEND: Special Educational Needs and Disabilities
SNS: sympathetic nervous system
SOS-S: Stress Overload Scale-Short
