# Supplementary material for: Breath-centered virtual mind-body medicine reduces COVID-related stress in women healthcare workers of the Regional Integrated Support for Education in Northern Ireland: a single group study
Source: Front Psychiatry. 2023 Jun 12;14:1199819. doi: 10.3389/fpsyt.2023.1199819 (PMC10291294; doi:10.3389/fpsyt.2023.1199819)
Supplement: Supplementary file 1 [file Data_Sheet_1.docx]

**Appendix A. PROGRAM EVALUATION CONSENT FORM**

**Breath-Mind-Body^TM^ Introductory Workshop Training for Stress Reduction**

**Background**

You are being asked to participate in this Program Evaluation because you have chosen to participate in the Breath-Mind-Body Introductory Workshop training being given by Dr. Richard P. Brown, Dr. Patricia Gerbarg, and their assistants to the staff of the Regional Integrated Support for Education Northern Ireland (RISE). This training program is designed to provide you with tools to use to decrease your own level of stress. Your participation in this evaluation will begin just before the training session when you will be asked to complete several questionnaires and will finish about 6 weeks after the training session when you return the third set of questionnaires.

**Purpose**

This Program evaluation will collect questionnaires before and after the training to assess the effect of the training on your experience of stress.

**What Happens In This Evaluation?**

You will be one of 40 participants in the BBM Introductory Workshop. The evaluation will be done by the Breath-Body-Mind team consisting of Dr. Patricia Gerbarg, Dr. Richard P. Brown, Dr. Vincent Conte, and Linda Lentini.

Three sets of questionnaires, one before the training, one after the training and one about 6 weeks after the training. Prior to the training, you will receive a Consent Form that explains the evaluation data collection, and privacy protection. You will be asked to sign and return the form to Linda Lentini. If you have any questions about the evaluation, you are welcome to ask Linda Lentini. Your participation in this evaluation will not affect your ability to participate in the training. The completion of each set of questionnaires will take 30-60 minutes. Five weeks after the training, you will receive the evaluation forms by e-mail. You will be asked to fill out the surveys and return them by email. If we do not receive the completed surveys by the end of the 6th week, you will be contacted by Linda Lentini. The purpose of her contact is to ask you fill out the forms over the phone or arrange for you to return them by mail, fax or email.

**Risks and Discomforts**

The risks of this evaluation are minimal. They include the time taken to fill out the questionnaires. The surveys have been chosen to avoid sensitive information.

There is always the risk that your answers to the questionnaires could be seen by someone other than the evaluation team. However, procedures are in place to protect the information obtained during the evaluation. These procedures include having all questionnaires marked with coded identification ID numbers not your name. Only Dr. Gerbarg and Dr. Brown will have access to the list that links your name with the coded ID number which will be kept in a file on a password protected computer. Your information will not be released without your permission unless required by law. No individual information will be given to your employer. Findings will be released in conglomerate form from the entire group, rather than individual reports.

You could find filling out the surveys stressful. If you need assistance Breath-Body-Mind staff will be available by e-mail or phone.

**Potential Benefits**

You will receive no direct benefit from your participation in this evaluation. However, you will have the personal satisfaction of knowing that your participation may help the Breath-Body-Mind team to better understand the efficacy of the training program and how to further improve it. You will also be contributing to the goals of the Breath-Body-Mind Foundation which intends to use the data collected in this evaluation for three purposes:

1. To write a report that can be used to support applications for grants to fund additional staff training in how to teach BBM practices to schoolteachers and students.

2. To write an article for publication to inform others of the potential benefits of this program.

3. To present the pooled data for teaching purposes.

**Confidentiality**

Information from the questionnaires may be used for evaluation purposes and may be published; however, your name will not be used in any publications.

**Your Rights**

Giving consent means that you have heard or read the information about this evaluation and that you agree to participate.

If at any time you decide to withdraw from this evaluation you will not suffer any penalty or lose any benefits to which you are entitled.

A member of the evaluation team will try to answer all of your questions. If you have questions or concerns at any time, or if you need to report an injury you may contact Linda Lentini, Felicity Dixon, or Natalie Tierney.

**Right to Refuse or Withdraw**

Taking part in this evaluation is voluntary. You have the right to refuse to take part in this evaluation. Your participation is completely up to you. Your decision will not affect your ability to complete the BBM program and it will not affect your employment. If you choose to take part, you have the right to stop at any time.

You signature indicates that you agree and consent to participate in this Program Evaluation of the Beath-Body-Mind Introductory Workshop Training.

Sign full name: _______________________________________ Date: _______________

Print your name: _____________________________________

Thank you for agreeing to participate in this Breath-Body-Mind Program Evaluation
