# Supplementary material for: Breath-centered virtual mind-body medicine reduces COVID-related stress in women healthcare workers of the Regional Integrated Support for Education in Northern Ireland: a single group study
Source: Front Psychiatry. 2023 Jun 12;14:1199819. doi: 10.3389/fpsyt.2023.1199819 (PMC10291294; doi:10.3389/fpsyt.2023.1199819)
Supplement: Supplementary file 2 [file Data_Sheet_2.docx]

**Appendix B. Table B1. Schedule of Breath-Body-Mind Introductory Course**

**RISE-NI December 16-18, 2020 9:00am–1:00 pm [EST]; 2:00pm-6:00pm [UK]**

| **UK** | **US EST** | **Day 1 Dec 16 Wed BBMIC RISE-NI** |
| --- | --- | --- |
| 2:00-2:15 | 9:00-9:15 | Welcome & Introduction. Assess Group, Discussion, Q & A. |
| 2:15-3:00  pm | 9:15-10:00  am | **Round I – Dr. Brown teaches BBM Practices:** Shaking, Ha Breath, 4-4 Breath, 4-4-6-2 Breath, Coherent Breathing, Relaxation Body Scan. Group Process, Discussion, Q & A |
| 3:00-3:15 | 10:00-10:15 | **Break 15 min** |
| 3:15-4:00 | 10:15-11:00 | **Round II – Dr. Brown teaches BBM Practices:** Tapping, Ha Breath, 4-4-6-2, Coherent Breathing, Breath Moving, Relax Body Scan-Open Focus Attention Training. Discussion |
| 4:00-4:15 | 11:00-11:15 | **Break 15 min** |
| 4:15-5:30 | 11:15-12:30 | **Group Process in Breakout Rooms (BORs)**  25 min Participants have 3 min each to introduce themselves, whatever they want to share.  50 min **Round III –** Teacher leads 4-4 arm circles, 10 min Coherent Breathing [observe and coach as needed] Discussion, Sharing, Q & A. Return to Main Room |
| 5:30-6:00 | 12:30-1:00 | Dr. Gerbarg reviews manual, homework, 5-Breath Tones. Discussion, Q & A |
| 6:00-6:30 | 1:00-1:30 | BBM Teachers meet in Faculty Breakout Room: Review of Day 1, share observations. |
| **UK** | **US EST** | **Day 2 Dec 17 Thursday BBMIC RISE-NI** |
| 2:00-3:00 pm | 9:00-10:00 am | Greet, Check-in, Assess Group  **Round I: Dr. Brown teaches BBM Practices:**  Tapping, HA, Love HA, 4-4-6-2, 4-4 Arm Circles, Coherent Breathing, Breath Moving, Relaxation Open Focus. Sharing, Discussion, Q & A |
| 3:00-3:15 | 10:00-10:15 | **Break 15 min** |
| 3:15-4:15 | 10:15-11:15 | **Round II: Dr. Brown teaches BBM Practices:**  Shake, 4-4-6-2, 4-4 Arm Circles with a Different Intention, Sky and Earth Energy Ball, Figure 8’s, Pearl of Light Coherent Breathing, Breath Moving, Relaxation Open Focus. Sharing Discussion |
| 4:15-4:30 | 11:15-11:30 | **Break 15 min** |
| 4:30-5:30 | 11:30-12:30 | **Lecture Part I – Dr. Gerbarg:** Neurophysiological effects of Breathing Practices on stress Response Systems, Sympatho-vagal Balance, Emotion Regulation, Social Engagement, Trauma Symptoms, and post-Traumatic Stress Disorder. Q & A |
| 5:30-6:00 | 12:30-1:00 | **Round III – Dr. Brown teaches**: Alternate Nostril Breathing. Discussion, Q & A |
| 6:00-6:30 | 1:00-1:30 | BBM Teachers meet in Faculty Breakout Room: Review of participants. |
| **UK** | **US EST** | **Day 3 Dec 18 Friday BBMIC RISE-NI** |

| 2:00-3:00 pm | 9:00-10:00 | Greet, Check-in, Assess Group  **Round I – Dr. Brown teaches BBM Practices:** Tapping, 4-4-6-2 Heart Focus. Great Harmonizer, Coherent Breathing + Breath Moving Heart Focus, Relaxation Open Focus Attention Training. Group Process Sharing, Discussion |
| --- | --- | --- |
| 3:00-3:15 | 10:00-10:15 | **Break 15 min** |
| 3:15-4:00 | 10:15-11:00 | **Round II – Dr. Brown teaches BBM Practices:** Ha Breath, 4 -4-6-2 **Heart Focus**, Coherent Breathing + Breath Moving **Heart Focus**, Relaxation **Open Focus Heart Focus** |
| 4:00-4:15 | 11:00 -11:15 | **Process: Heart’s Desire.**  Discussion. |
| 4:15-4:45 | 11:15-11:45 | **Lecture – Dr. Gerbarg – BBM for Children.** Videoclips illustrate BBM practices to help children with anxiety, trauma, withdrawal, sleep problems, anger outbursts, impulsive aggression, problems with attention, social relationships. Do CB with child video. Discussion, Q & A |
| 4:45-5:00 | 11:45-12:00 | **Break 15 min** |
| 5:00-5:30 | 12:00-12:30 | **Dr. Brown Ask Everyone to Write Their Responses to these Questions.**  What will you take away from this experience? How will you use what you have learned? Students discuss what they learned and how they intend to use it. |
| 5:30-5:45 | 12:30-12:45 | **Dr. Brown:** Instructions for Home Practices. Maintain Daily Practice.  **Dr. Gerbarg:** Follow-up sessions. Resources: Weekly Group Practices, Audio Tracks. Website. Newsletter. Teacher Training. Q & A Graduation Certificates |
| 5:45-6:00 | 12:45-1:00 | Closing, Song-Kong-Tong-Dong (vibrational vocalization with breath moving), Good-byes |
| 6:00-6:30 | 1:00-1:30 | **Teachers meet in Faculty Breakout Room:** Review participants and course. |

**Table B2. Description of the BBM Practices and Sequences**

| **Practices** | **Breath Rate & Pattern** | **Description of Practice** |
| --- | --- | --- |
| Shaking | Natural rhythm | Stand and shake body up and down |
| Ha Breath | 16-20 cpm for 10-20 breaths, rest 30 sec, repeat once or twice | Form 1: Sharp inhale as elbows draw back, palms upward.  Forceful exhale with loud sound of “Ha” as arms go forward, hands flick  Form 2: Sharp inhale as arms go up, hands open. Forceful exhale with “Ha” as elbows drop to waist, hands close.  Form 3: Love (Heart) Ha: Inhale more gently, elbows back, palms up. Gently extend arms forward with long, gentle ‘Ha”. |
| Tapping |  | Tap parts of the body in sequence with one or both hands from shoulders down to hands, chest down to feet and back up legs, torso |
| 4-4 Breath-Hum on Exhale | 5 cpm inhale=exhale | Gentle inhale 4-count, gentle exhale humming 4-count |
| Qigong 4-4-6-2 | 4 counts inhale, 4 hold, 6 exhale, 2 hold. Repeat x 10-16 | Inhale 4-count hands move up in front of body, arms extend upward, palms face ceiling, hold-4, exhale-6 as arms move slowly down to waist level palms up. Hold-2 |
| Painting the Waterfall | 5 cpm inhale=exhale | Imagine painting a waterfall Gently inhale-4, exhale-4  Back of hands stroke water up with inhale.  Palms stroke water down with exhale. |
| Qigong Sky and Earth | 5 cpm | Qigong movement. See Light Elixer Yoga, Qigong Master Robert Peng. 8 Cycles Video. www.robertpeng/store |
| Energy Ball – Pearl of Light | 5 cpm |  |
| Great Harmonizer of Breath | 5 cpm | Qigong movement. See BBM-IC Manual Attachment-C |
| Deep relaxing breaths | 2 slow deep breaths | Slow deep inhale. Slow exhale with a sigh. |
| Top-Down Muscle Relaxation | Natural rhythm | Soften muscle around eyes, face, neck, shoulders, back, chest, abdomen, arms, hands, legs, feet |
| Coherent Breathing 5 cpm | 5 cpm inhale=exhale | Gentle inhale 4-count, gentle exhale 4-count |
| Coherent Breathing Children | 5-6 cpm | Breath Buddy (small stuffed animal) sits on the child’s belly and is used toteach Coherent Breathing. Visualization. |
| Breath Moving | 5 cpm | During Coherent Breathing use the imagination to move the breath in circuits: 1) top of head-base of spine; 2) top of head-soles of feet |
| Bottom-up Body Scan | 5 cpm or natural rhythm | Place attention on feet, ankles, behind knees, hands, inside elbows, shoulders, inside abdomen, inside chest, throat, between eyes, top of head |
| Open Focus Attention Training increases flexibility between narrow and open focus.(Fehmi, Robbins 2008) | 5 cpm or natural rhythm | Alternate noticing of the space inside and outside various parts of the body, eg. mouth, ears, between eyes, arms, legs, abdomen, chest, throat. Notice space between thoughts or between emotions. |
